# Evaluation of Serologic Cross-Reactivity Between Dengue Virus and SARS-CoV-2 in Patients with Acute Febrile Illness — United States and Puerto Rico, April 2020–March 2021

**DOI:** 10.15585/mmwr.mm7110a3

**Published:** 2022-03-11

**Authors:** Jorge Munoz-Jordan, Jaime Cardona, Manuela Beltrán, Candimar Colón, Jarad Schiffer, Evelene Stewart-Clark, Briana Zellner, Vera Semenova, Yikun Li, Lily Tao Jia, Panagiotis Maniatis, Lucia Pawloski, Laura Adams, Gabriela Paz-Bailey, Vanessa Rivera-Amill, Freddy Medina

**Affiliations:** ^1^Division of Vector-Borne Diseases, National Center for Emerging and Zoonotic Infectious Diseases, CDC; ^2^Microbial Pathogenesis and Immune Response Laboratory, CDC; ^3^CDC COVID-19 Emergency Response Team; ^4^Ponce Research Institute, Ponce Health Sciences University, Ponce, Puerto Rico.

The diagnosis of dengue disease, caused by the dengue virus (DENV) (a flavivirus), often requires serologic testing during acute and early convalescent phases of the disease. Some symptoms of DENV infection, such as nonspecific fever, are similar to those caused by infection with SARS-CoV-2, the virus that causes COVID-19. In studies with few COVID-19 cases, positive DENV immunoglobulin M (IgM) results were reported with various serologic tests, indicating possible cross-reactivity in these tests for DENV and SARS-CoV-2 infections (*1*,*2*). DENV antibodies can cross-react with other flaviviruses, including Zika virus. To assess the potential cross-reactivity of SARS-CoV-2, DENV, and Zika virus IgM antibodies, serum specimens from 97 patients from Puerto Rico and 12 U.S.-based patients with confirmed COVID-19 were tested using the DENV Detect IgM Capture enzyme-linked immunosorbent assay (ELISA) (InBios International).* In addition, 122 serum specimens from patients with confirmed dengue and 121 from patients with confirmed Zika virus disease (all from Puerto Rico) were tested using the SARS-CoV-2 pan-Ig Spike Protein ELISA (CDC).^†^ Results obtained for DENV, Zika virus IgM, and SARS-CoV-2 antibodies indicated 98% test specificity and minimal levels of cross-reactivity between the two flaviviruses and SARS-CoV-2. These findings indicate that diagnoses of dengue or Zika virus diseases with the serological assays described in this report are not affected by COVID-19, nor do dengue or Zika virus diseases interfere with the diagnosis of COVID-19.

Persons infected with SARS-CoV-2 can be asymptomatic or experience a range of illnesses from mild fever to life-threatening respiratory disease. In mildly symptomatic patients with fever, COVID-19 might be confused with other diseases that have similar symptoms, including dengue and Zika virus diseases. Dengue, caused by four antigenically distinct dengue virus serotypes (DENV-1–4) transmitted by *Aedes spp. *mosquitoes, is usually a mild febrile illness but might evolve into severe dengue disease resulting in life-threatening conditions, such as dengue hemorrhagic fever and dengue shock syndrome. Dengue disease is a major public health problem throughout tropical and subtropical regions, causing approximately 400 million infections per year, 25% of which are clinically apparent (*3*). DENV-1–4 transmission has been reported in the Americas during the current COVID-19 pandemic, causing concerns about persons with COVID-19 antibodies being misdiagnosed based on results from a flavivirus antibody test because of antibody cross-reactivity. 

Laboratory diagnosis of dengue disease focuses on the detection of viral RNA by real-time reverse transcription–polymerase chain reaction (RT-PCR) or nonstructural protein 1 (NS1) antigen tests in blood specimens. These tests identify a large percentage of cases during the first few days of illness (*4*). After 5 days of illness, DENV-1–4 RNA and NS1 decline with the rise in antibody response; therefore, IgM antibody detection by ELISA becomes the primary option for diagnosing recent DENV-1–4 infections (*4*). Serologic cross-reactivity between DENV and Zika virus is an important limitation in the diagnosis of these diseases. In light of the overlapping symptoms associated with dengue disease and COVID-19, patients in areas where DENV-1–4 and SARS-CoV-2 circulate could be infected with either one of these viruses while they still have detectable levels of antibodies against the other. Patients might also have DENV-1–4 and SARS-CoV-2 coinfections. In addition, depending on the specificity of each test, a false positive serologic test result for one of the diseases is more likely during a period of low incidence if incidence of the other disease is high.

Recent reports indicated possible cross-reactivity in serologic (IgM) tests for DENV in specimens from confirmed COVID-19 cases (*1*,*2*). In a study of dengue disease cases detected before the COVID-19 pandemic, some specimens returned a false-positive result when tested for SARS-CoV-2 IgG or IgM. A study of 32 COVID-19 cases found no cross-reactivity with DENV, whereas only two of 44 dengue disease cases indicated cross-reactivity on a SARS-CoV-2 IgM ELISA (*3*). A more extensive evaluation of 11 SARS-CoV-2 immunochromatographic antibody tests indicated specificity in panels of 20–40 dengue specimens ranging from 85% to 100%, indicating variability of test performance (*5*). In another study, no cross-reactivity of dengue specimens in a SARS-CoV-2 IgM ELISA was observed, but cross-reactivity for SARS-CoV-2 in five of 26 confirmed Zika virus specimens did occur (*6*).

The purpose of this study was to assess the potential cross-reactivity of SARS-CoV-2 IgM antibodies in the DENV Detect IgM Capture ELISA, a Food and Drug Administration-approved ELISA test frequently used for the diagnosis of DENV-1–4 infections with demonstrated high sensitivity in the acute and early convalescent phases of the disease (*1*). A secondary aim was to determine whether Zika virus and DENV-1–4 IgM antibodies cross-react with the SARS-CoV-2 pan-Ig Spike Protein ELISA (*7*). Five serum specimen panels were evaluated; these included two panels from COVID-19 patients, one from dengue disease patients, one from Zika virus disease patients, and one from Zika virus and DENV-negative patients with acute febrile illness.

Since 2012, the Sentinel Enhanced Dengue Surveillance System in Puerto Rico has maintained a repository of serum and nasal swab specimens collected from febrile patients evaluated at several hospital-based acute febrile illness surveillance sites (*8*). A panel of 97 serum specimens obtained 4–9 days after illness onset from patients with confirmed COVID-19 (based on SARS-CoV-2 real-time RT-PCR positive test results)^§^ was prepared from specimens collected in Puerto Rico during April 2020–March 2021. A second panel consisted of 12 convalescent serum specimens from COVID-19 patients with high SARS-CoV-2 antibody titers collected in the United States during 2020–2021^¶^ and tested using the SARS-CoV-2 pan-Ig Spike Protein ELISA (*7*). To assess whether specimens from COVID-19 patients were cross-reactive with DENV IgM, these specimens were tested using the DENV Detect IgM Capture ELISA according to the manufacturer’s instructions. The remaining panels consisted of 365 specimens^**^ collected from patients with acute febrile illness in Puerto Rico before 2017; these specimens were evaluated as 1) DENV IgM-positive by the DENV Detect IgM Capture ELISA (122 specimens), 2) Zika virus IgM-positive by Zika virus MAC-ELISA (CDC) (122 specimens), and 3) both Zika virus and DENV IgM-negative (121 specimens). The DENV specimens were collected during 2012–2014; the Zika virus and acute febrile illness specimens were obtained during the 2016 Zika virus disease epidemic. Serum specimens were tested for SARS-CoV-2 antibodies using SARS-CoV-2 pan-Ig Spike Protein ELISA, as previously described (*7*), and were considered positive, negative, or equivocal according to their optical density ratio. All serum specimens used in this study were deidentified. This activity was reviewed by CDC and was conducted consistent with applicable federal law and CDC policy.^††^

None of the 97 specimens from COVID-19 patients collected in Puerto Rico tested positive for anti-DENV IgM; 95 specimens tested negative and two returned equivocal results, indicating a 100% specificity during the period of symptomatic disease when most patients with dengue disease or Zika virus disease are usually tested (Table). The convalescent serum specimens collected from 12 U.S. confirmed COVID-19 patients all tested negative. Among the 122 DENV IgM-positive specimens, two specimens returned positive anti–SARS-COV-2 pan-Ig test results. Similarly, two of 122 Zika virus IgM-positive and two of 121 negative specimens returned positive results, indicating a 98% specificity of the anti–SARS-CoV-2 Spike Protein pan-Ig ELISA.

**TABLE:** Cross-reactivity of SARS-CoV-2 in the DENV Detect IgM Capture ELISA* and of dengue virus and Zika virus in the CDC SARS-CoV-2 pan-Ig Spike Protein ELISA† — United States and Puerto Rico, April 2020–March 2021

Pathogen or syndrome	Location and collection time frame	Test	Analyte	No. of specimens	No. positive or reactive	No. negative or nonreactive	No. equivocal	% Specificity (95% CI)
**SARS-CoV-2**	Puerto Rico Dec 2020–Jan 2021	DENV Detect IgM Capture ELISA	Anti-DENV IgM	97	0	95	2	100 (96–100)
**SARS-CoV-2**	United States 2020–2021	DENV Detect IgM Capture ELISA	Anti-DENV IgM	12	0	12	0	100 (74–100)
**DENV**	Puerto Rico 2012–2014	SARS-CoV-2 pan-Ig Spike Protein ELISA	Anti-SARS-CoV-2 and total human antibodies	122	2	120	NA	98 (94–100)
**Zika virus**	Puerto Rico 2016	SARS-CoV-2 pan-Ig Spike Protein ELISA	Anti-SARS-CoV-2 and total human antibodies	122	2	120	NA	98 (94–100)
**Acute febrile illness**	Puerto Rico 2016	SARS-CoV-2 pan-Ig Spike Protein ELISA	Anti-SARS-CoV-2 and total human antibodies	121	2	119	NA	98 (94–100)

**Abbreviations:** DENV = dengue virus; ELISA = enzyme-linked immunosorbent assay; IgM = immunoglobulin M; NA = not applicable.

* http://inbios.com/wp-content/uploads/2016/05/900106-07-IVD-DENV-Detect-IgM-Capture-ELISA-Insert.pdf

^†^ https://www.biorxiv.org/content/10.1101/2020.04.24.057323v2

## Discussion

The results obtained for DENV and Zika virus IgM and SARS-CoV-2 antibodies evaluated with the tests described in this study indicated high specificity and minimal levels of cross-reactivity between the two flaviviruses (DENV and Zika virus) and SARS-CoV-2. A previous study reported a similar test specificity of the SARS-CoV-2 pan-Ig Spike Protein ELISA assay (99%) for pathogens unrelated to those evaluated in this study (*7*), and similarly high levels of specificity (97%) have been reported for the DENV Detect IgM Capture ELISA (*9*).

The findings in this report are subject to at least three limitations. First, the study was conducted with tests used at CDC laboratories for reference testing and do not constitute a direct assessment of other available tests. In addition, selection of specimens from acute and early convalescent phases of disease is based on the recommended time for dengue disease diagnosis; therefore, this study does not address cross-reactivity after day 9 of symptoms, when antibody levels might be higher than those detected during disease. The study did not assess cross-reactivity from COVID-19 vaccine-elicited antibodies. Finally, sampling in this study does not address the contribution of previously acquired IgG antibodies to the specificity of these tests.

These findings indicate that in a cohort of patients in Puerto Rico, where dengue disease is endemic, the serologic diagnosis of dengue disease with a commonly used IgM test is not affected by antibodies to SARS-CoV-2, nor do Zika virus and DENV IgM antibodies interfere with SARS-CoV-2 antibody detection. These results suggest that previously reported cross-reactivity between these viruses appears to be nonspecific and not a result of actual cross-reactivity from shared or similar epitopes. A possible explanation for these apparent cross-reactive results might be the presence of antibodies from a recent flavivirus infection in COVID-19 patients in areas of co-endemicity. Therefore, routine testing algorithms established for dengue and Zika diseases with the assays described in this report can proceed with the understanding that the chances of misdiagnosis of dengue or Zika virus diseases are not augmented by COVID-19, nor do dengue or Zika virus diseases interfere with the diagnosis of COVID-19.

**Summary**

**What is already known about this topic?**

In studies with few COVID-19 cases, positive dengue virus (DENV) immunoglobulin M results were reported with various serologic tests, indicating possible cross-reactivity in serologic tests for DENV and SARS-CoV-2 infections.

**What is added by this report?**

In a large cohort of febrile patients in Puerto Rico (where DENV is endemic) with recently confirmed SARS-CoV-2, DENV, or Zika virus infections, the specificity of DENV and SARS-CoV-2 enzyme-linked immunosorbent assays was ≥98%.

**What are the implications for public health practice?**

These findings indicate that diagnoses of dengue or Zika virus diseases with the serological assays described in this report are not affected by COVID-19, nor do dengue or Zika virus diseases interfere with the diagnosis of COVID-19.

## Footnotes

*
http://inbios.com/wp-content/uploads/2016/05/900106-07-IVD-DENV-Detect-IgM-Capture-ELISA-Insert.pdf

†
https://www.biorxiv.org/content/10.1101/2020.04.24.057323v2

§ Confirmed COVID-19 cases were based on tests conducted by Dengue Branch, Division of Vector-Borne Diseases, CDC.

¶ Twelve convalescent serum specimens from U.S. COVID-19 patients were tested at Microbial Pathogenesis and Immune Response Laboratory, CDC.

** These 365 specimens collected from patients with acute febrile illness in Puerto Rico before 2017 were evaluated at Dengue Branch, Division of Vector-Borne Diseases, CDC.

†† 45 C.F.R. part 46.102(l)(2), 21 C.F.R. part 56; 42 U.S.C. Sect. 241(d); 5 U.S.C. Sect. 552a; 44 U.S.C. Sect. 3501 et seq.
